# The effects of core training on athletic performance (speed and jumping) in different sports: a meta-analysis

**DOI:** 10.3389/fphys.2026.1946447

**Published:** 2026-10-02

**Authors:** İdris Kayantaş

**Affiliations:** Bingöl University, Bingöl, Türkiye

**Keywords:** core stability, core training, jumping performance, meta-analysis, speed performance

## Abstract

**Objectives:**

The aim of this study was to conduct a comprehensive, holistic examination, using meta-analysis, of the effects of core stabilisation training on speed and jumping-key indicators of athletic performance-in individuals participating in various sports.

**Methods:**

Studies published in Web of Science, PubMed and Google Scholar between 2020 and June 2026 examining speed and jumping performance were screened. Studies involving pre-test–post-test designs with experimental and control groups were recorded for analysis. A total of 49 studies meeting the inclusion criteria were included. This meta-analysis followed the PRISMA statement reporting guidelines. The CMA Ver. 2.0 statistical package was used to assess publication bias, calculate means and standard deviations, determine effect sizes and perform moderator analyses. The I² statistic assessed heterogeneity between studies; 25 per cent, 50 per cent and 75 per cent cut-off values represent low, moderate and high levels, respectively. Due to anticipated clinical and methodological diversity across sports disciplines, participant competitive levels, and test protocols, a random-effects model was applied *a priori* for all pooled analyses across speed and jumping outcomes. All estimates are reported with corresponding 95 per cent confidence intervals (CI). The α level was set at 0.05 for statistical significance.

**Results:**

The findings of this meta-analysis, which included a total of 49 studies, indicate that core training has a positive effect on the speed and jumping performance of individuals participating in various sports. The overall effect size calculated for speed performance was 0.601, whilst the overall effect size for jumping performance was determined to be 0.489. These findings indicate that core training has a positive effect on both speed and jumping performance.

**Conclusions:**

Core training is a useful complementary method for improving speed and jumping performance. Its effectiveness depends not only on training duration and sample size but also on exercise selection, intensity, frequency, athlete characteristics, and sport specificity. Therefore, combining core training with sport-specific sprint, strength, and plyometric exercises may enhance athletic performance.

## Introduction

The core, which acts as the central link in the kinetic chain, consists of the abdominal and spinal muscles, as well as the hip and diaphragm muscles (Rodriguez-Perea et al., 2023). The core plays a critical role in the efficiency of force transmission between the upper and lower limbs and, in particular, supports spinal stabilisation and postural control during dynamic movements (Zhang et al., 2025). The core constitutes the part of the human body referred to as its physical and functional ‘engine’. All movement originates from this central region and is then transmitted to the limbs. The core’s muscular structure differs fundamentally from that of the internal organs; for this region is frequently subjected to contraction and, as the point where all muscles work in unison, it develops a firm structure. From a theoretical perspective, a strong core enables force to be transferred efficiently and with minimal energy loss between different parts of the body (Luo et al., 2022).

Core exercises help to strengthen the body’s core in athletes, thereby improving balance, strength and flexibility (Hibbs et al., 2008). Core exercises are those that target the central core muscles and aim to develop these muscle groups in a balanced manner. These exercises are generally used to improve spinal stabilisation, support posture and enhance performance in athletes (Hodges et al., 1997). The active use of the core muscles enables athletes to move more quickly and with greater control on the field (Leetun et al., 2004).

In theory, a strong core enables the athlete to transfer the force generated from the ground through the lower body to the torso and, ultimately, to the upper body in the most efficient manner. It is thought that a weak or unstable core region negatively affects sporting performance by causing alterations in energy and force transmission, and increases the risk of injury in weak muscle groups (Sinha and Mili, 2021).

Various studies on core training-which is predominantly based on strengthening exercises—have shown that, in elite athletes across different sports, physical strength (core endurance and balance) and/or sport-specific athletic abilities (such as speed, strength and agility) improve with large and small effect sizes, respectively (Dong et al., 2023; Ozmen and Aydogmus, 2016). Although other researchers have reported specific benefits of core strengthening, they have not found sufficient evidence to confirm its effects on postural sway in athletes (Martins et al., 2019).

In recent years, various experiments have been carried out to investigate the effect of abdominal muscle training on athletic performance; however, the results are inconsistent and inconclusive. At the same time, most systematic reviews and meta-analyses have focused on the effects of basic training on injuries and illnesses (Stuber et al., 2014; Coulombe et al., 2017); however, to the best of our knowledge, there are no studies addressing its effects on athletes’ sport-specific performance. Therefore, a comprehensive review and quantitative analysis of this issue is required; this is the aim of this systematic review and meta-analysis. In this context, this study assessed the effect of general training on athletes’ athletic performance (e.g. exercise performance, agility, endurance and balance) and concluded that general training has a significant impact on athletes’ general endurance and balance, but has little effect on sport-specific performance. This is a systematic review and meta-analysis encompassing 25 studies that examined the effects of core training on balance, jumping performance and throwing/hitting performance. The results indicate that core training leads to significant improvements, particularly in balance and jumping performance; however, the effect may vary depending on the type of training (isolated or combined), its duration and participant characteristics (Rodriguez-Perea et al., 2023). It is reported that core training offers particular benefits in terms of postural stability and during the final stages of a sprint (Bartolomei et al., 2025).

In recent years, the role of neuromuscular training in athletic performance and the prevention of lower limb injuries has been the subject of increasing research. A systematic review conducted by Qin et al. (2024) demonstrated that integrated neuromuscular training significantly improves jumping performance in athletes and contributes to the reduction of lower limb injuries. In this review, which included a total of 19,805 athletes, an SMD of 0.45 (95% CI: 0.30–0.60) was reported for jumping performance and an SMD of 0.68 (95% CI: 0.62–0.74) for lower limb injuries; it was reported that programmes lasting at least 9 weeks and carried out 2–5 times a week yielded positive results. Furthermore, recent studies indicate that neuromuscular adaptations are not limited solely to general performance improvements, but that the training method employed and the specificity of the movements are also important. Wang et al. (2026) reported that unilateral flywheel-based complex training in male volleyball players led to more pronounced improvements, particularly in short-distance sprinting and change-of-direction performance, whilst Qin et al. (2026) noted that balance training led to significant improvements in dynamic balance—one of the performance indicators across different movement directions—in athletes with chronic ankle instability. Zou et al. (2026), however, noted that trampoline training had positive effects on balance performance in children and adolescents with special needs, but did not result in significant changes in lower limb strength. When these findings are considered together, it becomes apparent that the effects of neuromuscular training may vary depending on numerous factors, such as the training method, movement specificity, training dose, the targeted performance component and athlete characteristics. In this context, it may be considered more appropriate to view core training not as a sufficient method of performance development in its own right, but as a component of a broader strength and conditioning programme that supports force production and transmission, trunk stabilisation and neuromuscular coordination. In line with the findings presented in the literature, it is evident that, in modern athletic performance programmes, neuromuscular training is not aimed solely at developing a single physical capacity; rather, it should be approached as a combined strategy targeting various interrelated objectives, such as jumping performance, movement control and the reduction of injury risk.

This systematic review and meta-analysis was conducted with the aim of quantitatively examining the effects of core training-particularly on speed and jumping performance-across different sports disciplines, by evaluating new randomised intervention studies published in recent years, in addition to the findings reported in previous systematic reviews and meta-analyses. Whilst previous studies have examined the effects of core training on athletic performance within the broader context of performance outcomes, the present study focuses specifically on speed and jumping performance. For this reason, the aim of this study is not to replicate previous evidence syntheses, but to bring together the evidence from intervention studies published more recently, thereby providing an up-to-date and complementary quantitative assessment of these performance outcomes.

The decision to begin the literature review in 2020 was made in order to focus on recently published intervention studies and to evaluate the evidence that has accumulated since previous evidence syntheses. This time restriction does not imply that studies published prior to 2020 are scientifically invalid or insignificant; rather, as a significant proportion of these studies have already been addressed in previous evidence syntheses, it reflects the fact that the present review focuses on the evidence accumulated in the subsequent period.

The aim of this study was to comprehensively and holistically present, using a meta-analysis, the effects of core stabilisation training on speed and jumping-key indicators of athletic performance-among individuals participating in various sports.

## Methods

This meta-analysis was conducted in accordance with the PRISMA 2020 reporting guidelines (Page et al., 2020).

### Inclusion criteria

The inclusion criteria are as follows: a) The presence of an experimental and a control group; b) The experimental group must have undertaken core training and must have been drawn from a specific sport; c) The control group must have undergone some form of training d) Studies must have been published between 2020 and June 2026 e) Studies must contain appropriate data (arithmetic mean, standard deviation, sample size, sport discipline, etc.) f) Studies must include at least one of the following tests: speed or jump tests.

### Exclusion criteria

The exclusion criteria are as follows: a) Studies that do not meet the inclusion criteria for this study; b) Studies lasting less than 4 weeks; c) Studies for which access permission has not been granted; d) Studies that are not based on experimental results and are not original; e) Studies that do not contain appropriate data; e) Reviews, seminar and conference abstracts.

### Data extraction

The data extraction form was prepared in accordance with the Cochrane Handbook for Systematic Reviews of Interventions (Higgins et al., 2021). Data relating to the included studies were recorded separately on the data extraction form by two independent researchers. The data obtained by the researchers were then compared, and any entries where discrepancies were found were re-evaluated. To prevent unit-of-analysis dependency and the artificial underestimation of standard errors arising from multiple test measurements within the same cohort, a pre-defined outcome selection hierarchy was established: when a study reported multiple sprint splits (e.g., 10 m, 20 m, 30 m), the 20 m sprint split was prioritized for speed performance; for jumping performance, Countermovement Jump (CMJ) height was prioritized over other jump variations (such as standing long jump or squat jump). When multiple independent intervention arms were compared against a single control group within the same study, sample sizes of the control group were divided proportionally across comparisons to ensure statistical independence.

Disagreements were resolved through discussion, and where necessary, the opinion of a third researcher was sought. Once consensus had been reached, data from studies meeting the inclusion criteria were included in this meta-analysis.

### Data collection and selection criteria

A literature review was conducted in June 2026 using the Google Scholar, PubMed and Web of Science databases. During the search process, keywords identified in line with the research question and inclusion criteria were used. The search results revealed a total of 24,033 records: 13,913 in Web of Science, 670 in PubMed and 9,450 in Google Scholar. As a result of the title screening, 315 records deemed relevant to the research topic were recorded for further assessment. Duplicate studies were checked amongst these records, and duplicate records were removed to prevent the same study from being assessed more than once. Following the removal of duplicate records, the abstracts of the remaining studies were reviewed, and those found to be relevant to the research question were selected for full-text assessment.

A literature review was conducted to identify randomised controlled trials investigating the effects of core training on speed (10, 15, 20, 30 and 40 m) and jumping (vertical jump and standing long jump) performance. The search strategy utilised the terms “core training”, “core exercise” and “core stability” – which define core training – alongside the keywords “speed”, “sprint” and “jump”, which describe speed and jumping performance. These terms were combined as follows using the Boolean operators OR and AND: (“core training” OR “core exercise” OR “core stability”) AND (speed OR sprint OR jump). The search was restricted to studies published in 2020 and thereafter.

No prospective protocol for this systematic review and meta-analysis was registered on PROSPERO or other systematic review registry platforms. The full database-specific search strategy utilized the following Boolean search string across Web of Science, PubMed, and Google Scholar: (“core training” OR “core exercise” OR “core stability”) AND (speed OR sprint OR jump).

Following a review of titles and abstracts and an assessment of full-text eligibility, 49 studies that met the pre-defined inclusion criteria were included in the meta-analysis. Data relating to speed were analysed in 34 of these studies, whilst data relating to jumping performance were analysed in 36. The process of identifying and selecting studies is presented in **Figure 1** in accordance with the PRISMA 2020 (Page et al., 2020) reporting guidelines.

**Figure 1:**
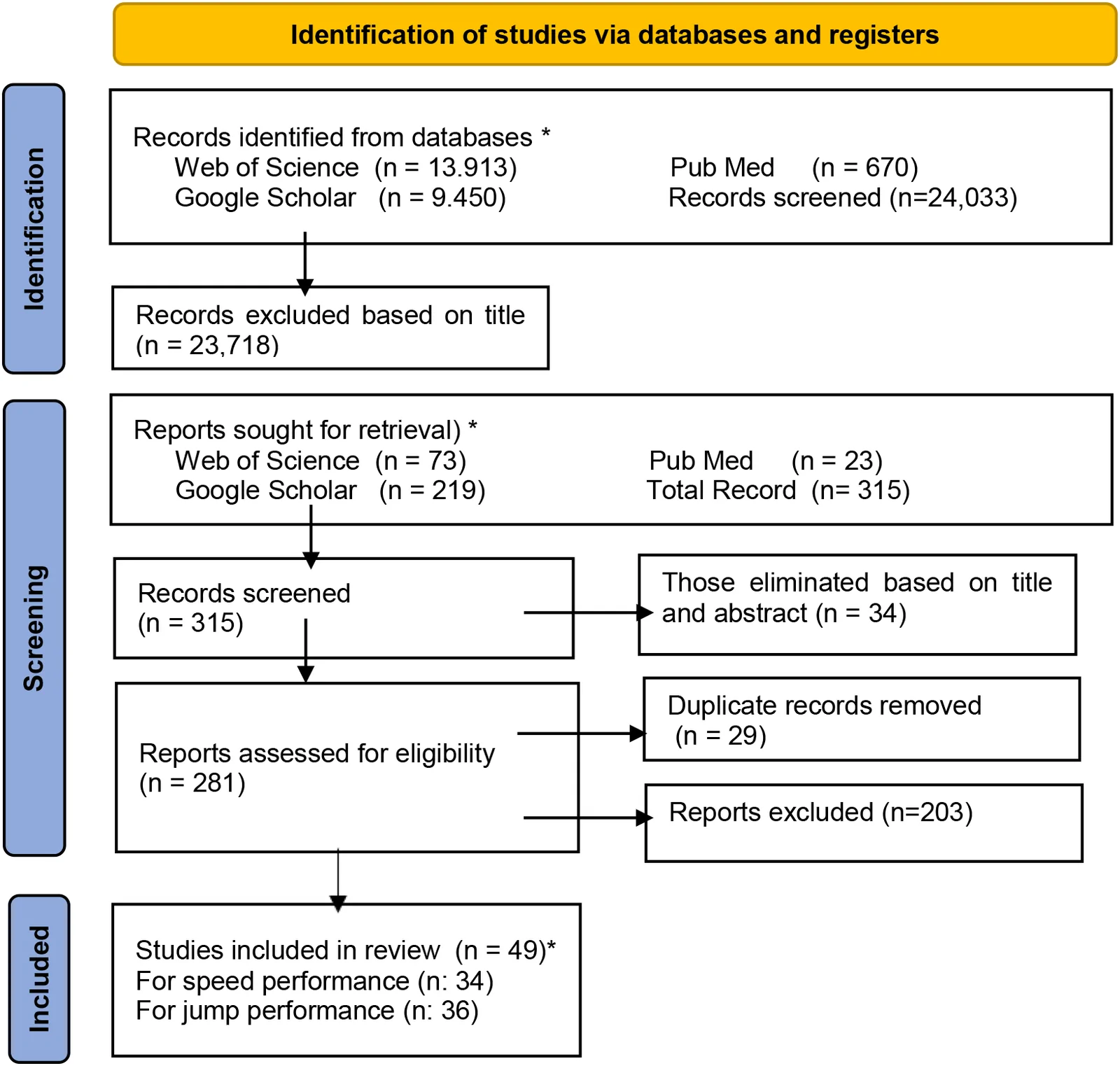
PRISMA 2020 flow diagram. *As some studies assessed both sprint and jump performance, these studies have been included in both categories. Consequently, the combined total of sprint (n = 34) and jump (n = 36) studies (n = 70) appears higher than the total number of studies included in the review (n = 49).

### Statistical analyses

For various outcome data, the Comprehensive Meta Analysis Version 2 (CMA Ver. 2.0) statistical package (Borenstein et al., 2005) was used to analyse publication bias, means, standard deviations, effect sizes and moderator analyses. The I² statistic was used to assess the heterogeneity between studies; the 25 per cent, 50 per cent and 75 per cent cut-off values represent low, moderate and high levels, respectively. Due to the expected clinical and methodological differences between the studies in terms of sample characteristics, sports disciplines, intervention durations and performance tests, a random-effects model was used in this study. The random-effects model takes into account both the actual differences in effect between studies and the variation arising from sampling error.

All estimates are reported together with their corresponding 95 per cent confidence intervals (CI). The α level was set at 0.05 to indicate statistical significance. Cohen’s classification was used in the interpretation of effect sizes. Cohen’s d is an effect size index that measures the difference between the means of two groups in terms of standard deviation units. The classification, established by Cohen (1992) and widely accepted in statistics, is as follows:


Small Effect:(d= 0.20), Medium Effect:(d=0.50), Large Effect:(d=0.80)

Due to inherent clinical and methodological diversity across sports disciplines, participant competitive levels, intervention protocols, and performance testing modalities, a random-effects model (DerSimonian-Laird) was implemented *a priori* across all primary analyses rather than relying on arbitrary I² decision cut-offs. Sprint performance is evaluated via completion time (seconds), where a reduction indicates enhanced performance, whereas jumping performance is evaluated via height or distance, where an increase indicates improvement. To ensure directional harmonization across all outcomes, sprint time reductions were directionally harmonized (time decreases coded positively) so that positive effect sizes (SMD > 0/Hedges’ g > 0) consistently denote performance enhancement favouring the intervention group across both sprint and jump parameters. Standardized mean differences were calculated based on pre-to-post change scores standardized by post-test standard deviations using Hedges’ g formulation:


ΔM=Mpost−Mpre

$$\text{SMD}=\frac{\text{ΔME}-\Delta \text{MC}}{\text{SDpost}}$$

where ΔME represents the mean pre-to-post change in the experimental group, ΔMC represents the change in the control group, and SDpost denotes the pooled standard deviation of the post-test scores. Hedges’ g was calculated by applying the small-sample bias correction factor J to the SMD estimates.

## Results

### Results relating to the speed characteristic

The findings regarding publication bias for the speed variable, the heterogeneity test, the effect sizes from individual studies, and the moderator analysis are presented below.

#### Findings on publication bias relating to speed

The funnel plot illustrating publication bias and the results of the Orwin Protection Number test are shown below.

As shown in **Figure 2**, the vast majority of studies fall within the funnel and are clustered around the average effect size. In a funnel plot, the studies are expected to show a symmetrical distribution around the effect size. However, it cannot be said that there is perfect symmetry in the funnel plot. The findings from the funnel plot are not recommended for assessing publication bias on their own. They must be evaluated in conjunction with other tests for publication bias. The test results for the Orwin-protected N are presented below.

**Figure 2:**
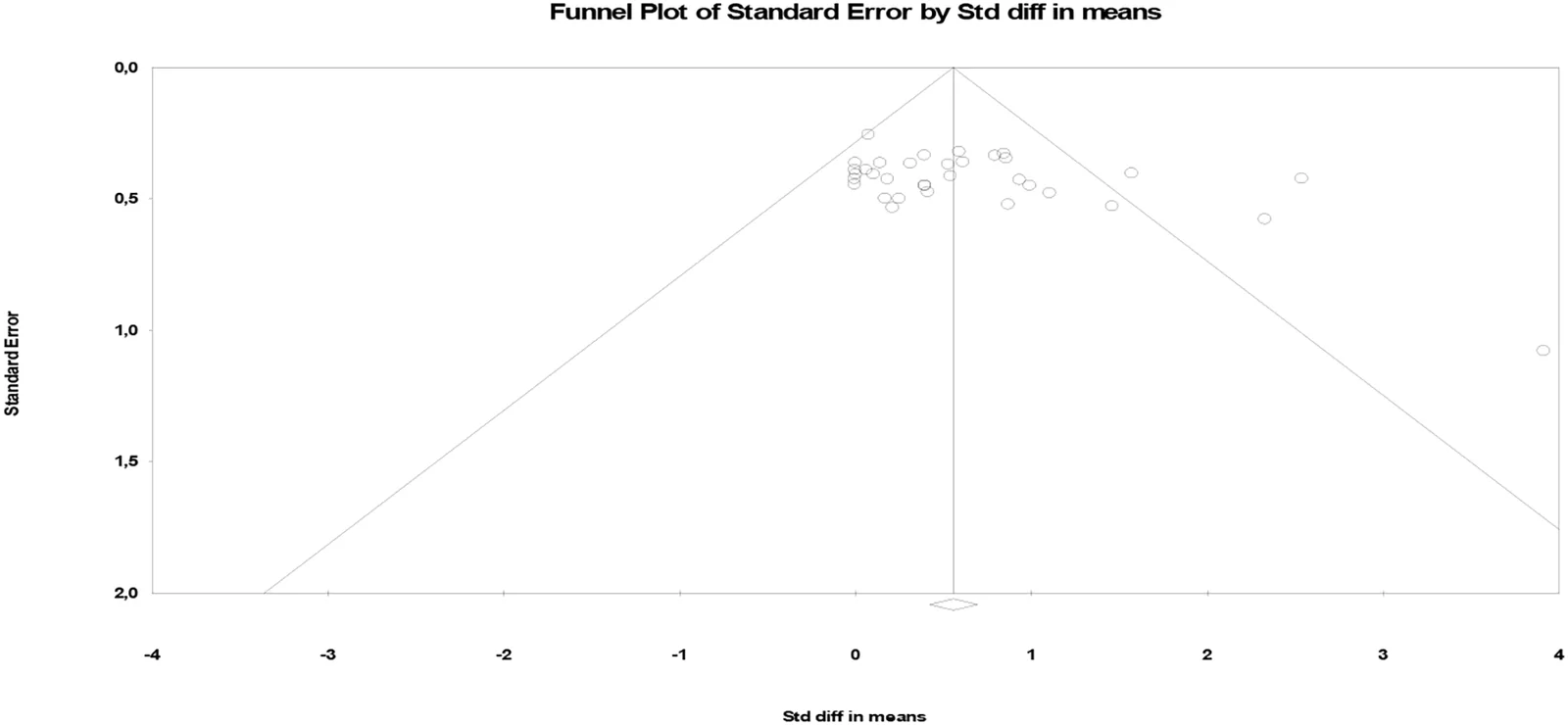
Funnel chart illustrating publication bias.

Rosenthal’s Classic Fail-Safe N analysis shows that 34 studies were included in the meta-analysis and that approximately 614 further studies showing no effect would be required to negate the statistical significance of the observed effect. This figure is well above the threshold of 5k + 10 = 180 proposed by Rosenthal. It can therefore be concluded that the findings of the meta-analysis are robust against publication bias and that the results provide strong evidence. However, because Fail-Safe N alone is insufficient to exclude potential reporting bias or small-study effects, further statistical assessments using Egger’s linear regression and trim-and-fill analyses were conducted (**Tables 1** and **2**).

**Table 1:** Egger's regression intercept.

Variable	Value
Intercept	3,000
Standard error	1,178
95% lower limit (2-tailed)	0,599
95% upper limit (2-tailed)	5,401
t-value	2,545
df	32,000
P-value (1-tailed)	0,007
P-value (2-tailed)	0,015

**Table 2:** Duval and Tweedie’s trim and fill.

	ST (n)	Fixed effects			Random effects			Q Value
		PE	LL	UL	PE	LL	UL	
Observed values		0,556	0,419	0,692	0,601	0,390	0,812	75,590
Adjusted values	4	0,642	0,513	0,771	0,686	0,481	0,892	90,039

The results of the Egger regression test analysis showed that the intercept coefficient was 3.000. The standard error of the intercept was 1.178, the t-value was 2.545 and the degrees of freedom were 32. The two-tailed significance level was calculated as p = 0.015, which is less than the 0.05 significance level. Accordingly, it has been concluded that the constant term is statistically significant. Furthermore, the fact that the 95 per cent confidence interval for the constant term lies between 0.599 and 5.401 and does not include zero supports the statistical significance of the result obtained. This statistically significant intercept (p = 0.015) indicates the presence of funnel plot asymmetry for sprint performance. Rather than definitively establishing publication bias, this asymmetry may reflect small-study effects or genuine between-study heterogeneity across different sporting disciplines and sprint testing protocols.

Duval and Tweedie’s trim-and-fill analysis estimated that four studies might be missing. Under the random-effects model, the observed pooled effect size was 0.601 (95% CI: 0.390–[upper limit]), whilst the effect size adjusted using the trim-and-fill method was found to be 0.687 (95% CI: 0.482–[upper limit]). The fact that the positive pooled effect size persisted following the correction indicates that the studies presumed to be missing had no significant moderating effect on the overall finding.

The Egger regression test indicated statistically significant funnel plot asymmetry (intercept = 3.000, SE = 1.178, t = 2.545, df = 32, p = 0.015; 95% CI: 0.599 to 5.401; **Table 1**). Duval and Tweedie’s trim-and-fill method imputed four potentially missing studies on the left side of the distribution, resulting in an adjusted random-effects effect size of 0.686 (95% CI: 0.481 to 0.892; **Table 2**). The presence of significant funnel plot asymmetry suggests the potential influence of small-study effects or reporting bias; however, this cannot be definitively attributed to publication bias alone, as substantial between-study clinical and methodological heterogeneity likely contributed to the observed distribution. The Orwin’s fail-safe N analysis indicated that 614 additional missing studies would be required to bring the p-value above the significance level, suggesting that the observed findings are relatively robust to potential publication bias (**Table 3**).

**Table 3:** Results for Orwin’s fail-safe N.

Classic Fail-Safe N	
Z-Value for Observed Studies	8,554
P-Value for Observed Studies	0,000
Alpha	0,050
Tails	2,000
Zfor alpha	1,959
Number of Observed Studies	34,000
Number of missing Studies that would bring P-Value t > alpha	614,000

#### Results on the magnitudes of effects related to the speed characteristic

A total of 34 studies were included in the meta-analysis. A statistically significant level of heterogeneity was observed among the studies (Q = 75.90; df = 33; p < 0.000; I² = 56.343 per cent). In accordance with our *a priori* statistical strategy accounting for clinical and methodological diversity across sports disciplines, the random-effects model was utilized as the primary model. In the random-effects model, the effect size (ES) was calculated as 0.601 (95% CI: 0.390–0.812; Z = 5.586; p < 0.000), and it was determined that the intervention had a moderate, statistically significant positive effect. This result indicates that core training has a positive effect on speed performance.

An examination of **Figure 3** reveals that, in individual studies, the effect size ranged from a maximum of 3.919 (Werasirirat et al., 2022) to a minimum of 0.000 (Genç and Ciğerci, 2020; Gottlieb et al., 2025). It is observed that core training has a small effect size in 16 studies, a moderate effect size in 6 studies, and a high effect size on speed in 12 studies.

**Figure 3:**
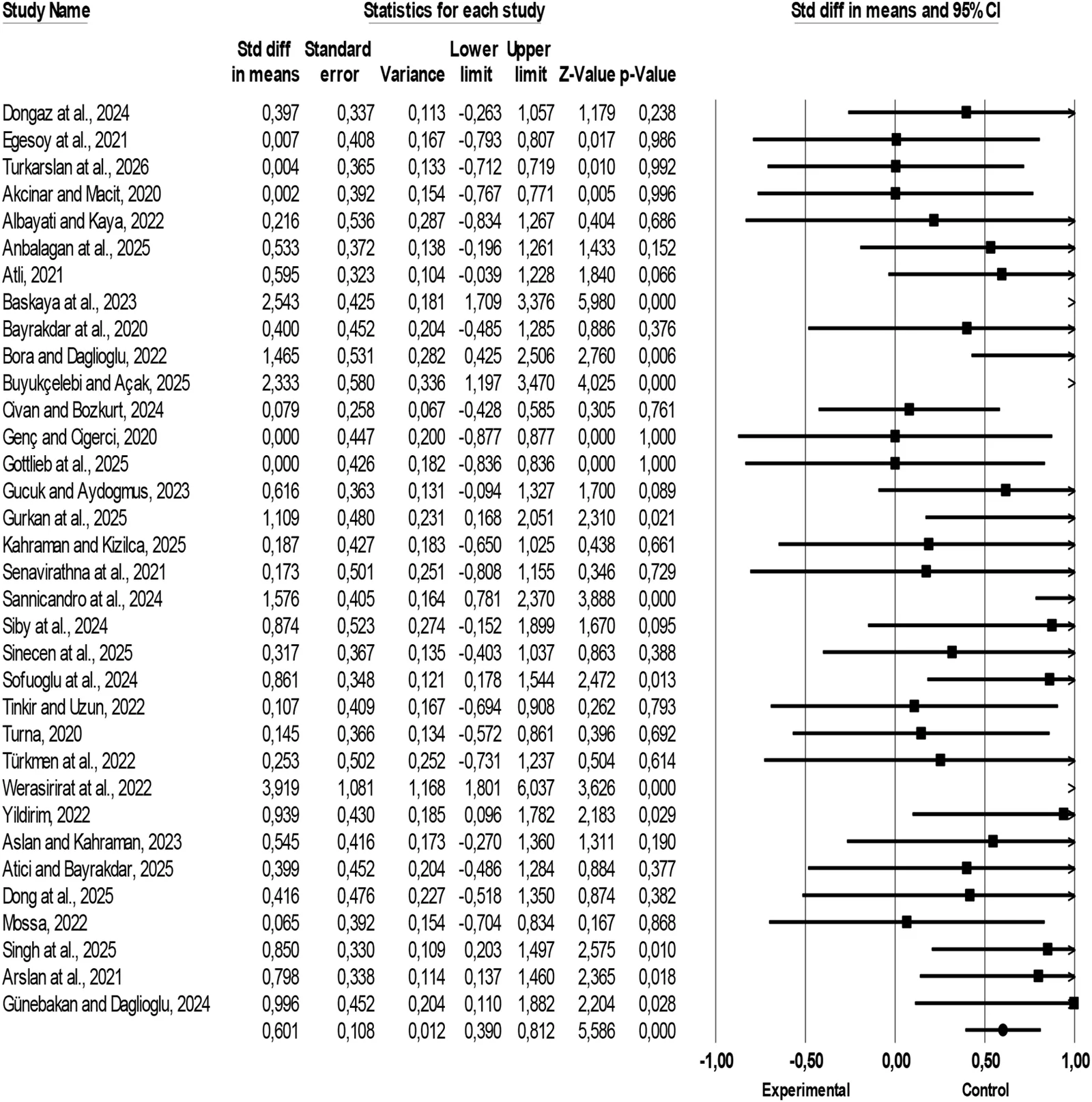
Scatter plot of individual studies on speed performance.

#### Results of the moderator analysis for the speed variable

As there was a disproportionate distribution of the number of individual studies across disciplines and speed categories, no subgroup analysis was carried out. Meta-regression analyses were conducted for the moderators of sample size and year of publication. The findings illustrating the relationship between sample size and effect size are presented in **Figure 4** below.

**Figure 4:**
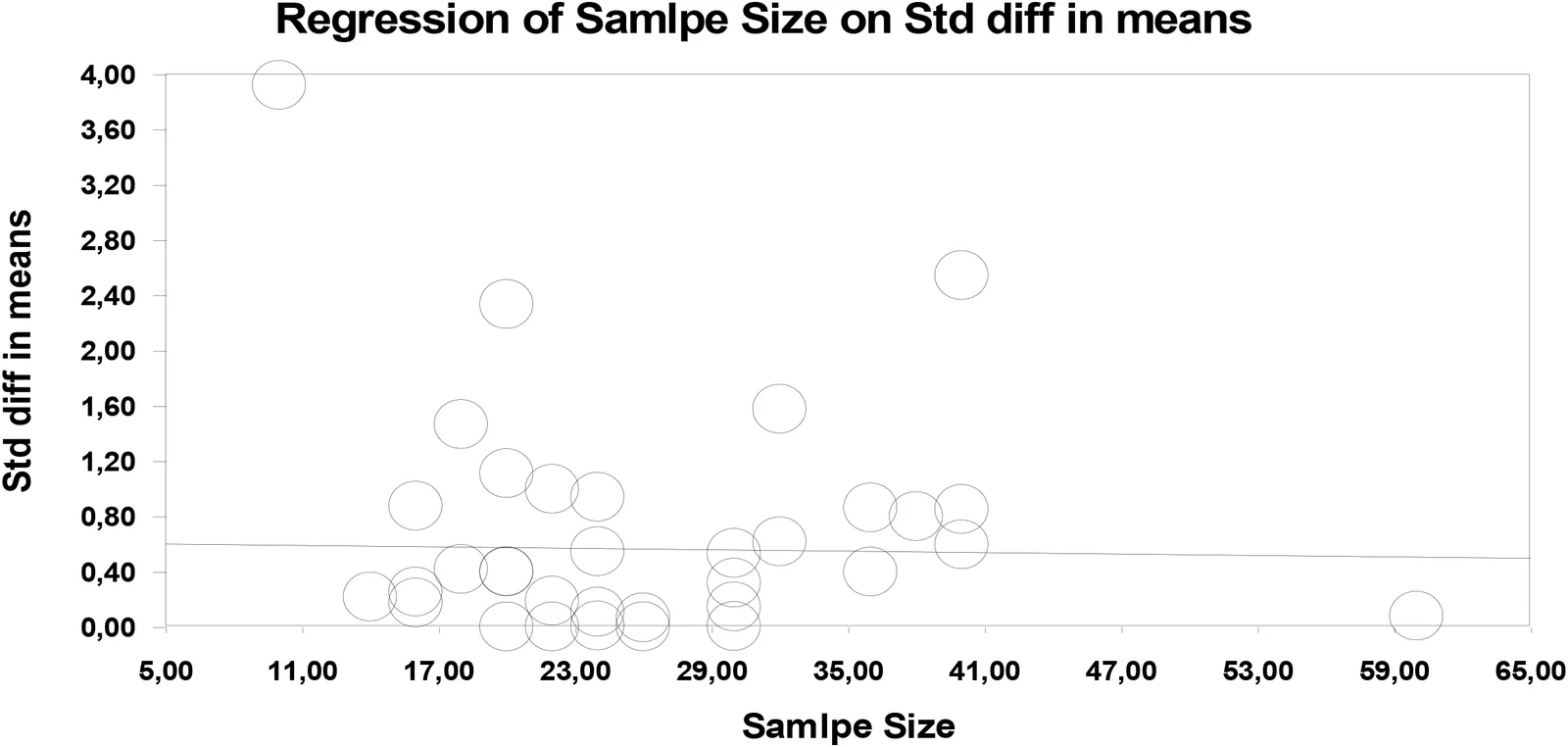
Meta-regression results illustrating the relationship between effect size and sample size.

On examining **Figure 4**, it can be seen that there is a slight decrease in the effect size as the sample size increases. Further statistical findings relating to this decrease are presented in **Table 4** below.

**Table 4:** Meta-regression results illustrating the relationship between effect size and sample size.

Term	PE	SE	LL	UL	Z-value	P-value
Slope	-0,001	0,006	-0,013	0,010	-0,275	0,783
İntercept	0,607	0,199	0,217	0,997	3,052	0,002

PE, Point Estimate; SE, Standard Error; LL, Lower Limit; UP, Upper Limit.

An examination of **Table 4** reveals that no statistically significant relationship was found between the year of publication and effect size (β = −0.001; SE = 0.006; Z = −0.275; p = 0.783). The fact that the slope coefficient is close to zero and that the 95 per cent confidence interval includes zero (−0.013 to 0.010) indicates that the year of publication did not significantly affect the effect size. These findings indicate that the year in which the studies were published is not a significant moderator of the intervention’s effect size. This result is also consistent with the meta-regression plot we shared earlier; the slight slope observed in the plot is not statistically significant (p = 0.783). Findings regarding the effect size in relation to another moderator, the year of publication, are presented in **Figure 5**.

**Figure 5:**
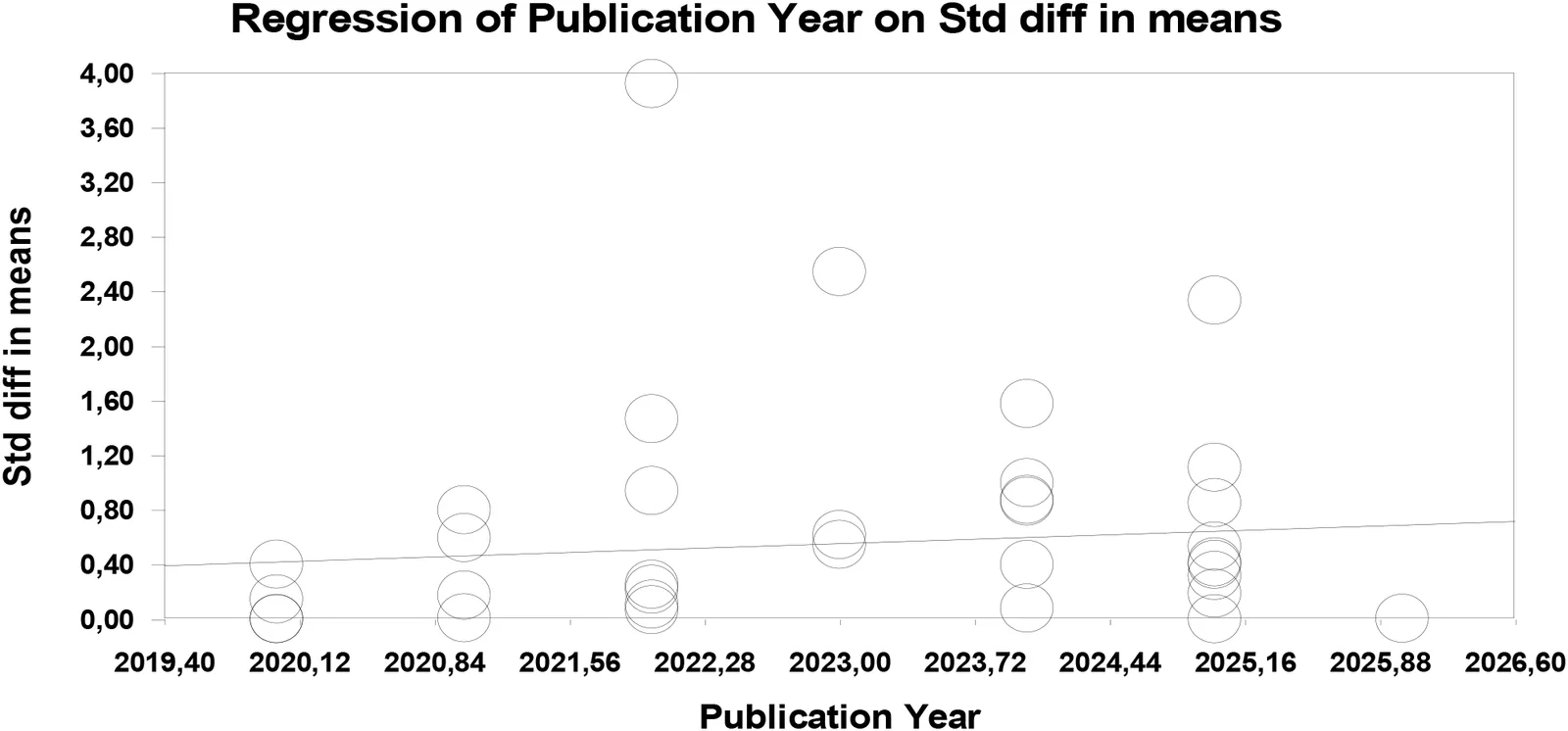
Meta-regression showing effect size by publication year.

Although a slight positive trend was observed between publication year and effect size in the meta-regression analysis, it was found that the relationship was weak and that considerable heterogeneity persisted across the studies. The graph suggests that the year of publication does not explain the effect size to a statistically significant extent. However, for a definitive interpretation, the meta-regression coefficient (β), the 95 per cent confidence interval and the p-value must be assessed together. The data for these values are presented in **Table 5**.

**Table 5:** Meta-regression results showing the relationship between effect size and year of publication.

Term	PE	SE	LL	UL	Z-value	P-value
Slope	0,044	0,038	-0,030	0,120	1,168	0,242
İntercept	-90,333	77,756	-242,733	62,067	-1,161	0,245

PE, Point Estimate; SE, Standard Error; LL, Lower Limit; UP, Upper Limit.

An examination of **Table 5** reveals that no statistically significant relationship was found between the year of publication and effect size (β = 0.044; SE = 0.038; Z = 1.169; p = 0.242). Although the slope coefficient is positive, the fact that the 95 per cent confidence interval includes zero (–0.030 to 0.120) indicates that the year of publication did not significantly affect the effect size. These findings indicate that the year of publication is not a significant moderator variable explaining the effect of the intervention.

### Results of the meta-analysis on jumping performance

The publication bias associated with the jump effect, the heterogeneity test, the effect sizes from individual studies, and the findings of the moderator analysis are presented below.

#### Findings on publication bias relating to the jumping feature

The funnel plot illustrating publication bias and the results of the Orwin Protection Number test are shown below.

As shown in **Figure 6**, the vast majority of studies fall within the funnel and are clustered around the average effect size. In a funnel plot, studies are expected to show a symmetrical distribution around the effect size. It can be said that there is symmetry in the funnel plot. The findings from the funnel plot are not recommended for assessing publication bias on their own. They should be evaluated in conjunction with other tests for publication bias. The test results for the Orwin-protected N are presented below.

**Figure 6:**
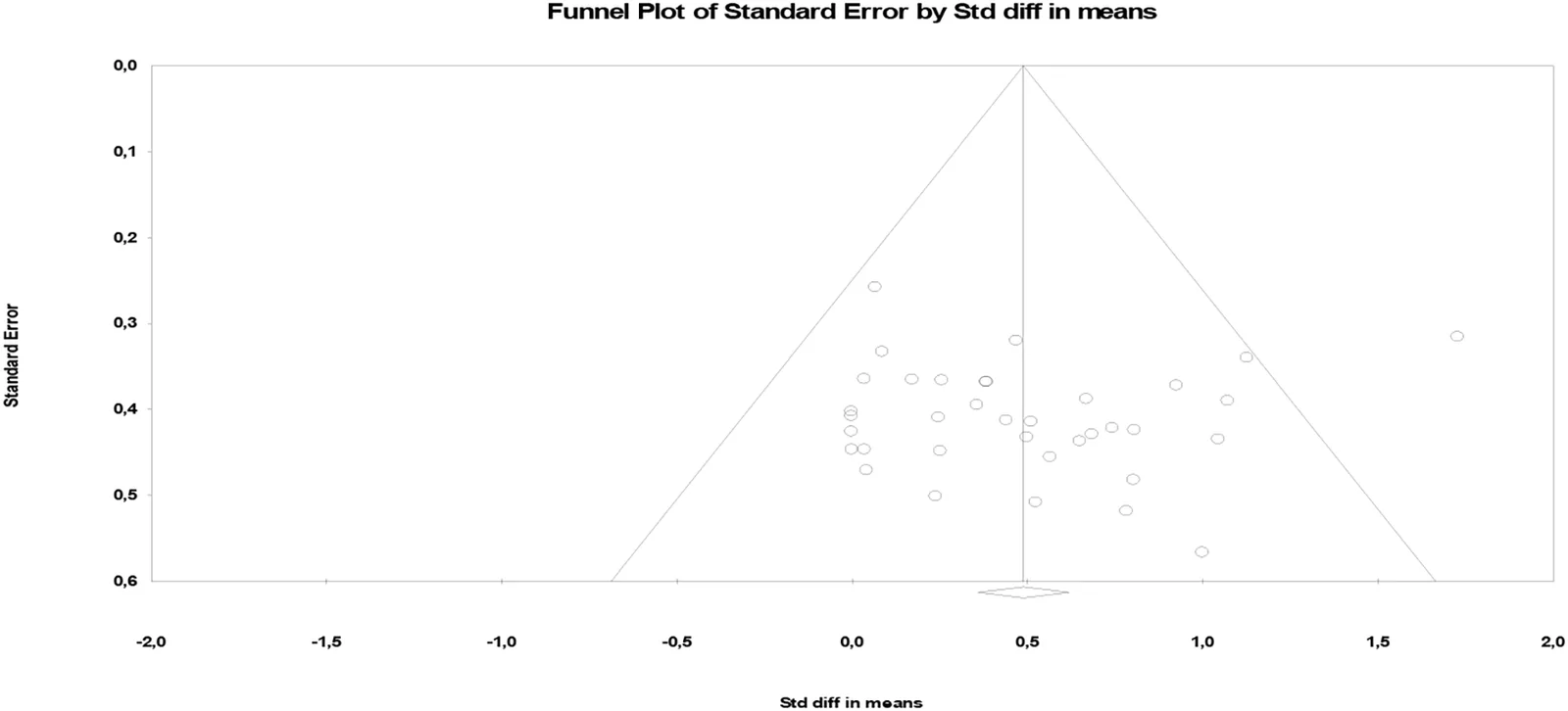
Findings from the funnel plot for the jump property.

Rosenthal’s Classic Fail-Safe N analysis shows that 36 studies were included in the meta-analysis and that approximately 461 further studies showing no effect would be required to negate the statistical significance of the observed effect. This value is well above the threshold of 5k+10 = 180 proposed by Rosenthal. It can therefore be concluded that the findings of the meta-analysis are robust against publication bias and that the results provide strong evidence. However, to comprehensively evaluate potential small-study effects beyond the Fail-Safe N metric, Egger’s regression and trim-and-fill analyses were also examined for jumping outcomes (**Tables 6** and **7**).

**Table 6:** Egger's regression intercept.

Variable	Value
Intercept	0,108
Standard error	1,175
95% lower limit (2-tailed)	-2,281
95% upper limit (2-tailed)	2,497
t-value	0,091
df	34,000
P-value (1-tailed)	0,463
P-value (2-tailed)	0,927

**Table 7:** Duval and Tweedie’s trim and fill.

	ST (n)	Fixed effects			Random effects			Value
		PE	LL	UL	PE	LL	UL	
Observed Values		0,488	0,358	0,618	0,489	0,344	0,633	42,548
Adjusted Values	2	0,514	0,387	0,641	0,516	0,373	0,658	45,786

ST, Studies Trimmed; PE, Point Estimate; LL, Lower Limit; UP, Upper Limit.

The Egger regression test showed that the asymmetry in the funnel plot was not statistically significant (intercept = 0.108, SE = 1.176, t = 0.092, df = 34, p = 0.927). The fact that the 95% confidence interval (-2.281–2.497) includes the value zero also supports this finding. Accordingly, no statistically significant funnel plot asymmetry was detected amongst the studies included in the meta-analysis. Consequently, the findings do not provide sufficient statistical evidence to suggest the presence of small-study effects or any associated potential publication bias. The results of the heterogeneity analysis for jumping performance are presented in **Table 8**.

**Table 8:** Results of the heterogeneity test.

Model	Studies(n)	Effect size and 95% confidence interval					Test of null (2-Tail)		Heterogeneity			
		PE	SE	Var.	LL	UP	z	p	Q	Df	P	I^2^
Fixed	34	0,556	0,070	0,005	0,420	0,693	7,995	0,000	75,90	33	0,000	56,343
Random	34	0,601	0,108	0,012	0,390	0,812	5,586	0,000				

PE, Point Estimate; SE, Standard Error, Var., Variance; LL, Lower Limit; UP, Upper Limit.

The impact of potential missing studies on jumping performance was evaluated using Duval and Tweedie’s trim-and-fill method. The analysis imputed two potentially missing studies on the left side of the distribution. Under the primary random-effects model, the observed point estimate of 0.489 (95% CI: 0.344 to 0.633) adjusted minimally to 0.516 (95% CI: 0.373 to 0.658; **Table 7**). Because the Egger regression test indicated no significant asymmetry (intercept = 0.108, SE = 1.175, t= 0.091, df= 34, p = 0.927; 95% CI: -2.281 to 2.497; **Table 6**) and the trim-and-fill adjustment produced only a negligible shift in effect magnitude while preserving statistical significance, these findings indicate minimal evidence of small-study effects or systematic reporting bias regarding jumping performance.

A total of 36 studies were evaluated for jumping performance. Heterogeneity analysis revealed low between-study variance (Q = 42.548; df = 35; p = 0.178; I² = 17.741%). Under the *a priori* random-effects model, the overall pooled effect size was calculated as SMD = 0.489 (95% CI: 0.345 to 0.634; Z = 6.631; p < 0.001; **Table 9**), demonstrating that core training had a moderate, statistically significant positive effect on jumping performance and that the findings were consistent across studies.

**Table 9:** Results of the heterogeneity test.

Model	Studies(n)	Effect size and 95% confidence interval					Test of null (2-Tail)		Heterogeneity			
		PE	SE	Var.	LL	UP	z	p	Q	Df	P	I^2^
Fixed	36	0,488	0,066	0,004	0,358	0,618	7,358	0,000	42,548	35	0,178	17,741
Random	36	0,489	0,074	0,005	0,345	0,634	6,631	0,000				

PE, Point Estimate; SE, Standard Error, Var.; Variance; LL, Lower Limit; UP, Upper Limit.

An examination of **Figure 7** reveals that, in individual studies, the effect size ranged from a maximum of 1.729 (Uslu et al., 2025) to a minimum of 0.000 (Arslan and Ergin, 2022; Gottlieb et al., 2025; Hassan et al., 2023). It is observed that core training has a small effect size in 3 studies, a moderate effect size in 15 studies, and a high effect size on jumping performance in 18 studies.

**Figure 7:**
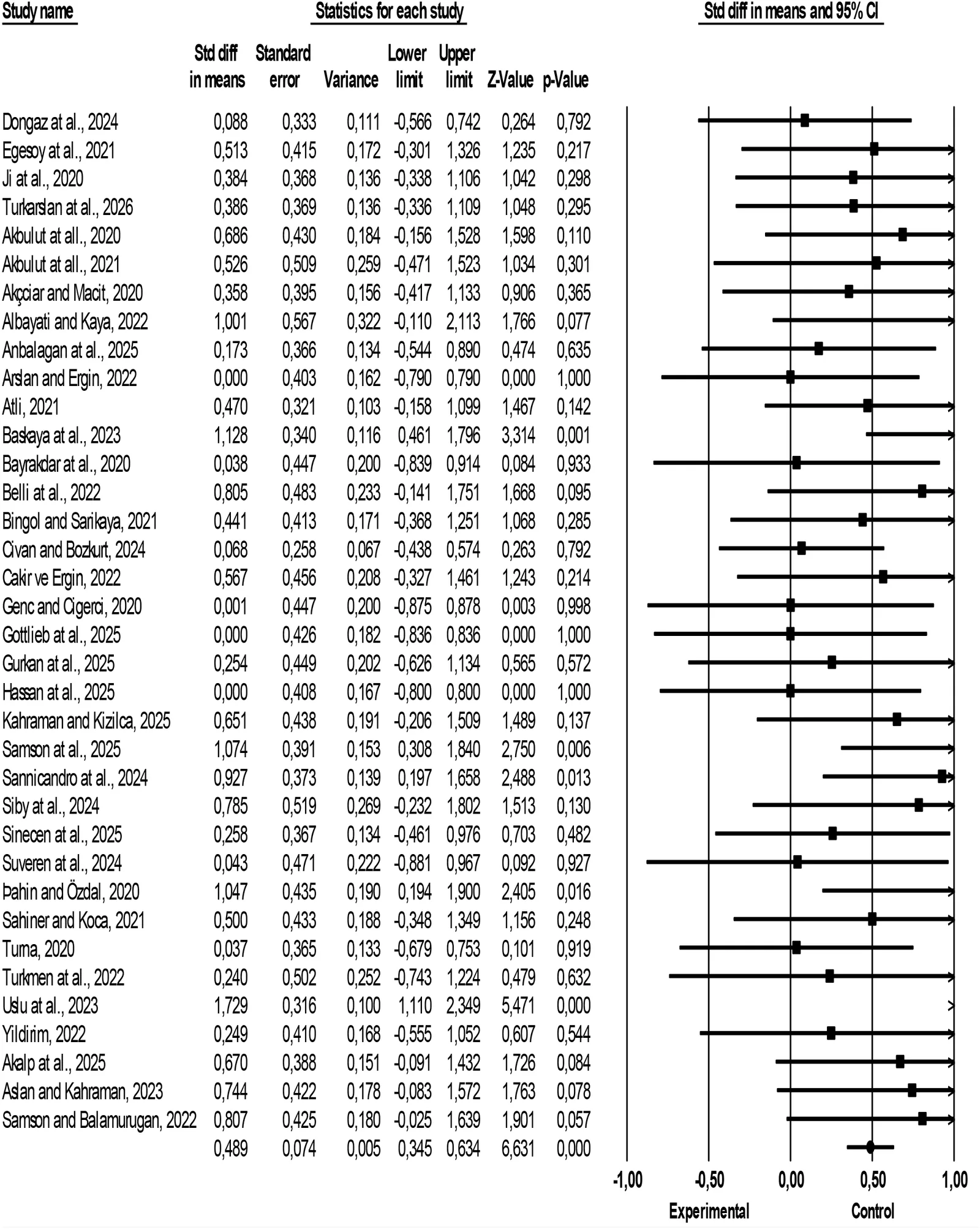
Scatter plot of individual studies on the jumping characteristic.

#### Results of the moderator analysis

The findings regarding the year of publication and effect size are presented in **Figure 8**.

**Figure 8:**
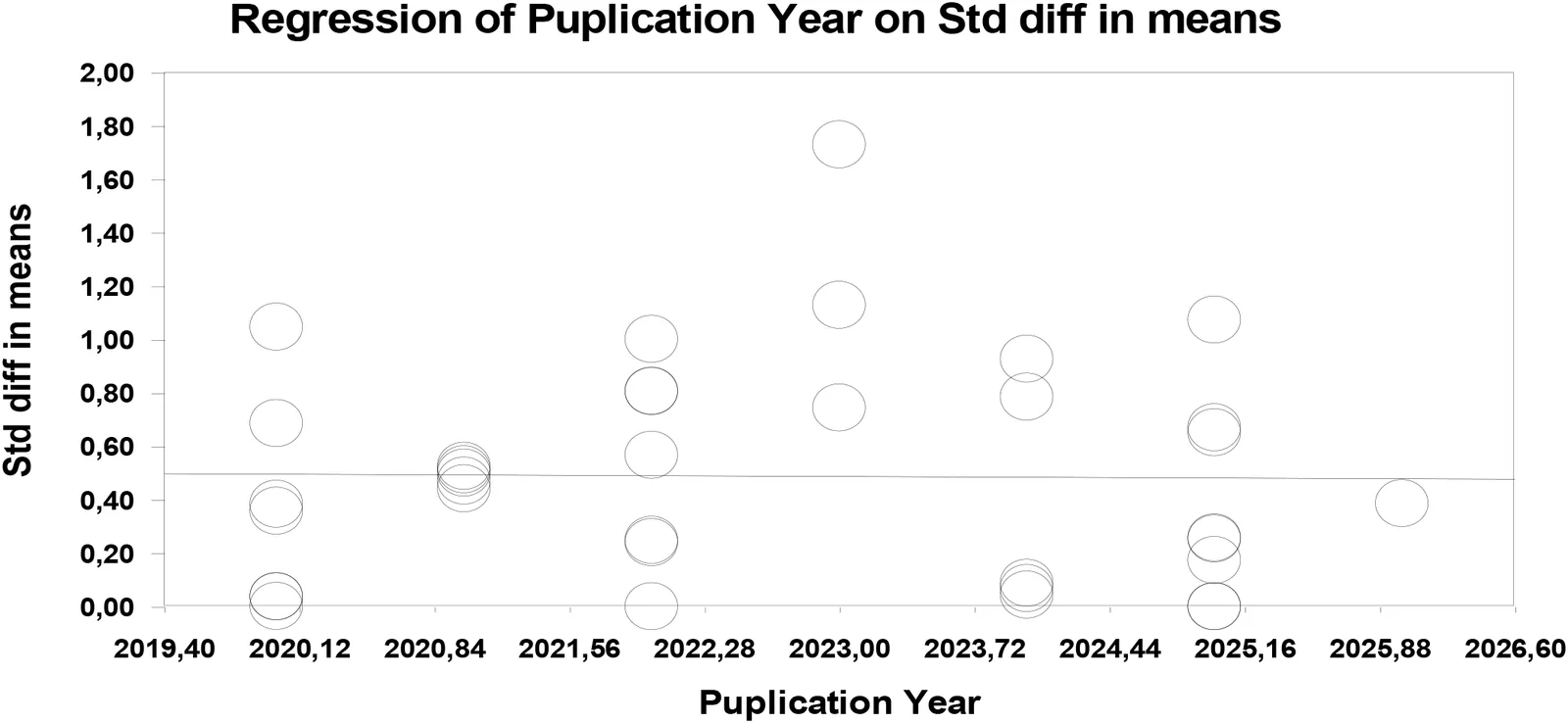
Meta-regression results showing the relationship between effect size and year of publication.

In the meta-regression analysis conducted by year of publication, no significant trend in effect sizes over time was observed. This finding indicates that the effect of core training on performance outcomes does not vary depending on the period in which the research was conducted. The data for these values are presented in **Table 10**.

**Table 10:** Meta-regression results showing the relationship between effect size and year of publication.

Term	PE	SE	LL	UL	Z-value	P-value
Slope	-0,002	0,034	-0,071	0,065	-0,080	0,935
İntercept	6,194	07,515	-132,013	144,402	0,087	0,930

PE, Point Estimate; SE, Standard Error; LL, Lower Limit; UP, Upper Limit.

In the meta-regression analysis conducted to assess the moderating effect of publication year on effect size, no statistically significant relationship (p = 0.935) was found between publication year and effect size (β = −0.002; SE = 0.034; Z = −0.081; p = 0.936). The fact that the 95 per cent confidence interval includes zero (−0.071 to 0.065) indicates that the year of publication did not significantly affect effect size. These findings indicate that there is no significant moderator variable explaining the effect of the year of publication. The findings regarding effect size in relation to sample size, another moderator, are presented in **Figure 9**.

**Figure 9:**
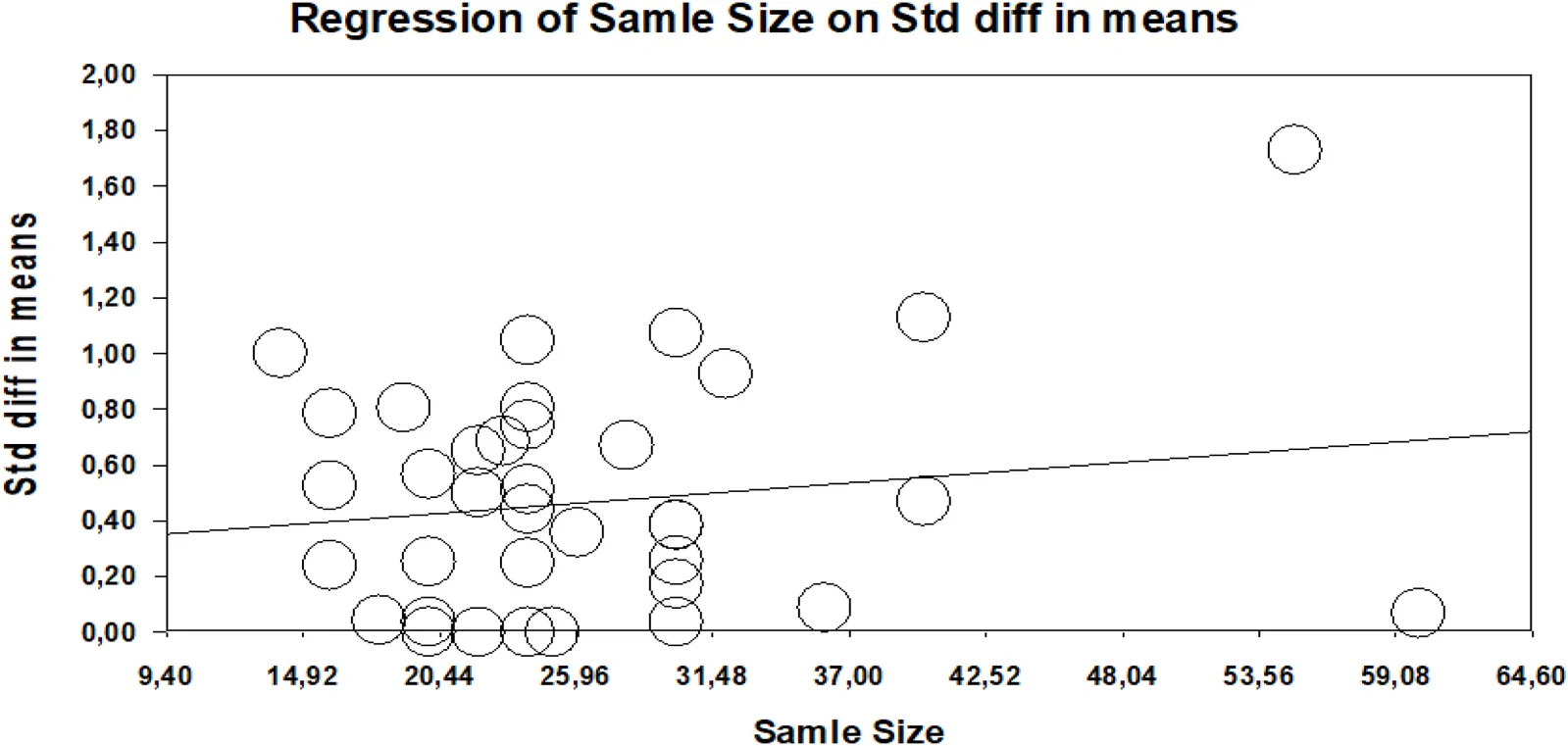
Meta-regression results showing the association between effect size and sample size.

In the meta-regression analysis, a slight positive trend in effect sizes was observed as sample size increased. However, the low regression slope indicates that sample size explains only a limited degree of the variation in effect size. The fact that the studies show a wide range of effect sizes across different sample sizes suggests that factors other than sample size may also play a significant role in the impact of core training on performance outcomes. The data for these values are presented in **Table 11**.

**Table 11:** Meta-regression results showing the association between effect size and sample size.

Term	PE	SE	LL	UL	Z-value	P-value
Slope	0,006	0,005	-0,004	0,017	1,171	0,241
İntercept	0,290	0,181	-0,066	0,646	1,596	0,110

PE, Point Estimate; SE, Standard Error; LL, Lower Limit; UP, Upper Limit.

In the meta-regression analysis, no statistically significant relationship (p = 0.241) was found between sample size and effect size (β = 0.006; SE = 0.005; Z = 1.171; p = 0.241). The fact that the 95 per cent confidence interval includes zero (–0.004 to 0.017) indicates that sample size does not significantly affect effect size. These findings indicate that sample size is not a significant moderator of the intervention effect. Findings regarding the effect size in relation to training duration, another moderator, are presented in **Figure 10**. The results of the Orwin’s fail-safe N analysis for jumping performance are presented in **Table 12**.

**Figure 10:**
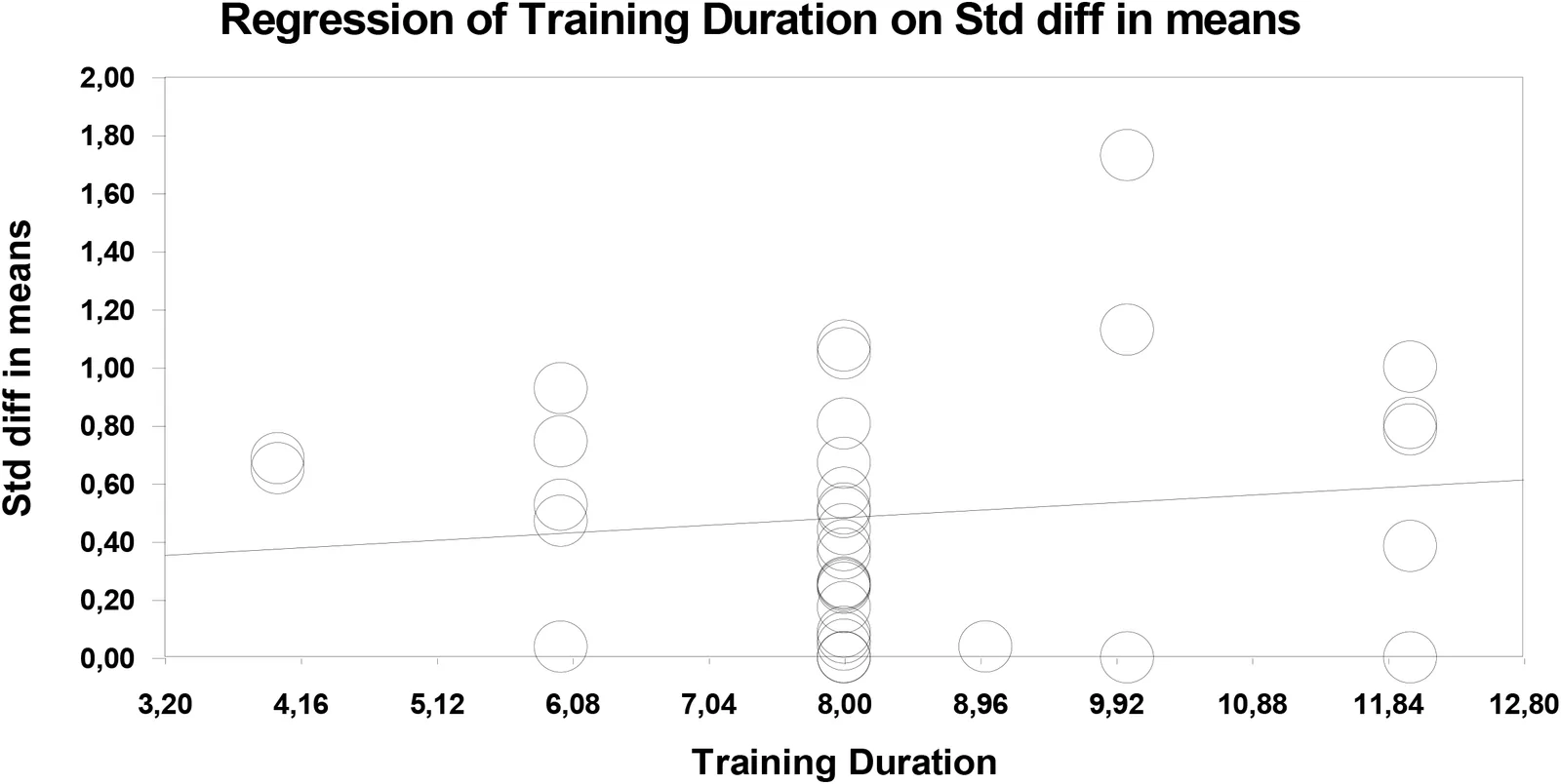
Meta-regression results for effect size and training duration.

In the meta-regression analysis, a slight positive trend was observed between training duration and effect size; however, it is considered that this relationship is weak and that training duration plays only a limited role in explaining the heterogeneity between studies. For this reason, training duration is not considered, on its own, to be a moderator that significantly influences the effect size. The data for these values are presented in **Table 13**.

**Table 12:** Results for Orwin’s fail-safe N.

Classic Fail-Safe N	
Z-Value for Observed Studies	7,281
P-Value for Observed Studies	0,000
Alpha	0,050
Tails	2,000
Zfor alpha	1,959
Number of Observed Studies	36,000
Number of missing Studies that would bring P-Value t > alpha	461,000

**Table 13:** Meta-regression results on effect size and training duration.

Term	PE	SE	LL	UL	Z-Value	P-Value
Slope	0,027	0,035	-0,042	0,096	0,767	0,442
İntercept	0,266	0,296	-0,314	0,847	0,898	0,369

PE, Point Estimate; SE, Standard Error; LL, Lower Limit; UP, Upper Limit.

In the meta-regression analysis conducted to assess the moderating effect of training duration on effect size, no statistically significant relationship was found between training duration and effect size (p = 0.442) (β = 0.027; SE = 0.035; Z = 0.767; p = 0.442). The fact that the 95 per cent confidence interval includes zero (–0.042 to 0.096) indicates that training duration does not significantly affect effect size. These findings indicate that there is no significant moderator variable explaining the effect of training duration on the intervention. Methodological quality and risk of bias across the included trials were formally assessed using the Cochrane Risk of Bias 2 (RoB 2) tool and visualized via the robvis package (McGuinness and Higgins, 2020; **Figure 11**). Across the 70 outcome evaluations, the majority of trials (90.0%, n = 63) were judged as having ‘some concerns’, 8.6% (n = 6) as ‘high risk’, and 1.4% (n = 1) as ‘low risk’. The primary driver of ‘some concerns’ was Domain 2 (deviations from intended interventions) due to the inherent logistical impossibility of blinding participants and personnel in exercise and strength training protocols. Conversely, high methodological strength was demonstrated in Domain 4 (measurement of the outcome, 90.0% low risk) and Domain 3 (missing outcome data, 95.7% low risk), reflecting the high objectivity of automated timing gates and jump mats used across the included trials.

**Figure 11:**
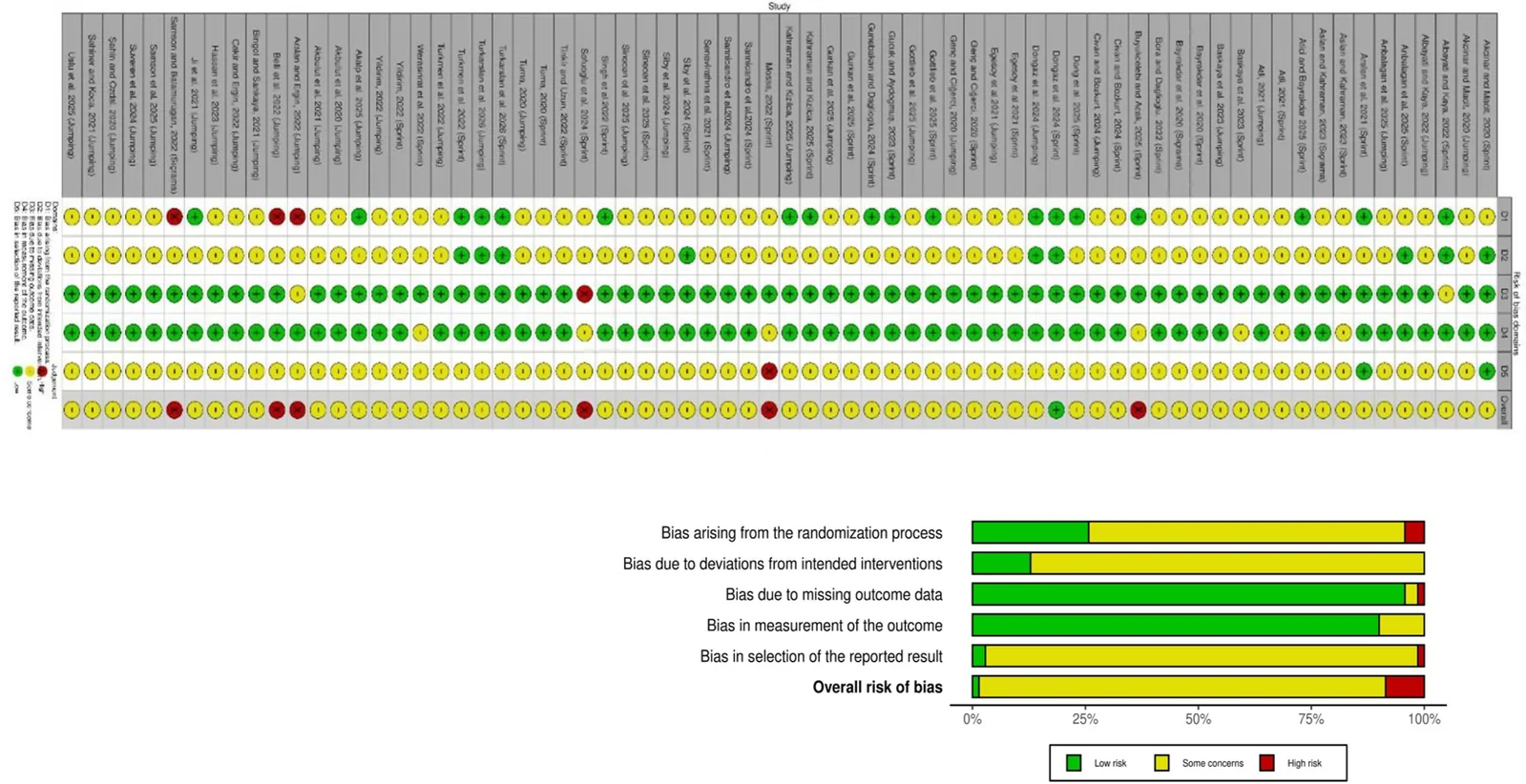
Assessments of methodological quality and risk of bias (RoB 2) for studies conducted in the field of sports performance outcomes (jumping and sprinting).

## Discussion

When the results of this study are considered alongside the findings of previous research, it is evident that core training generally has a positive effect on the speed and jumping performance of individuals participating in various sports. However, it is evident that the findings in the literature are not entirely consistent, and that the effect of core training on performance may vary depending on various factors, such as the athletes’ age and performance level, training history, the duration and frequency of the programme, exercise content, training intensity and sport-specific training.

### The effect of core training on sprint performance

One of the core region’s key roles in sporting performance is to facilitate the transfer of force between the limbs and to maintain trunk stability during movement. The effective transfer of force generated by the lower limbs through the trunk during a sprint is crucial for acceleration and sprint performance. It is therefore thought that developing core strength and stability may help to enhance acceleration capacity, particularly in short-distance sprint performance.

The positive effects of core training on sprint performance have been demonstrated in studies conducted across various sports. Arslan and Akkuş (2025) reported that a 12-week core training programme administered to male amateur footballers significantly improved their 10-metre and 30-metre sprint performance, as well as their anaerobic power. Similarly, Sannicandro et al. (2020) demonstrated that core training administered to basketball players led to a significant improvement in 10-metre sprint performance. Hoshikawa et al. (2013) reported an improvement in sprint performance following core stabilisation training administered to young footballers aged 12–13, in addition to their regular football training. In a study conducted by Boyacı and Afyon (2017), the researchers found that a 12-week core training programme resulted in significant improvements in 20-metre sprint performance.

These positive results are also supported by the findings of systematic reviews and meta-analyses. Luo et al. (2023) reported that core training may have positive effects on physical performance indicators, such as sprint performance, in footballers. A study conducted by Sofuoğlu et al. (2024) also demonstrated that a core stabilisation programme implemented in young footballers improved their 20-metre sprint performance. These results suggest that the contribution of core training to sprint performance cannot be explained solely by an increase in trunk strength; improving trunk stability and the more effective transfer of force between the lower limbs and the trunk may also play a role in this improvement.

However, it is evident that the findings regarding sprint performance do not point in the same direction across all studies. Baykara et al. (2024) reported that, whilst core-based training improved jumping performance in young taekwondo athletes, it did not result in a significant change in sprint performance. Similarly, Parkhouse and Ball (2011) found no significant improvement in sprint performance following a 6-week core exercise programme. These findings suggest that core training alone may not lead to a direct improvement in sprint performance.

These varying results in sprint performance can be explained by a number of factors. Sprint performance is not solely dependent on core strength and stability; it is influenced by many components, such as maximum lower-limb strength, explosive strength, sprint technique, neuromuscular coordination and movement economy. In addition, the type and intensity of the core exercises performed, the duration and weekly frequency of training, the athletes’ age and training history, and the sprint distance used may also influence the results. Furthermore, the degree to which core exercises are specific to the sprint movement is also significant. For this reason, it is thought that combining core exercises with sport-specific sprint training may be a more effective approach.

### The effect of core training on jumping performance

Improving core stability can contribute to jumping ability—one of the key indicators of explosive performance by enabling more effective force transmission along the kinetic chain. In particular, the effective transfer of force generated by the lower limbs through the trunk and the maintenance of trunk stability are important for performance during vertical jumps. It is therefore thought that core stability and strength training may support jumping performance (Zemkova and Zapletalova, 2022; Cabrejas et al., 2022).

There are numerous studies in the literature demonstrating the positive effects of core training on jumping performance. Akalp et al. (2025) reported that, following an 8-week combined core training programme, the experimental group showed an 11.2 per cent improvement in vertical jump performance. Similarly, Mahmoud (2018) found that a 10-week core exercise programme, carried out twice a week, significantly improved the standing long jump and vertical jump performance of young footballers. Doğan et al. (2016) reported an improvement in vertical jump performance following an 8-week core training programme administered to footballers. A study conducted by Boyacı and Bıyıklı (2018) on male footballers aged 11–13 also demonstrated that a 10-week core training programme improved jumping performance.

Studies conducted in various sports also support these positive findings. Bilici and Selçuk (2018) reported that a 10-week core training programme implemented with volleyball players aged 14–16 improved their vertical jump performance. Sharma et al. (2012) demonstrated a significant increase in vertical jump performance following a 9-week core training programme administered to university volleyball players. A study conducted by Şengür and Yılmaz (2021) on female athletes also reported that core training led to improvements in vertical jump performance in favour of the experimental group. Furthermore, Sannicandro et al. (2020) demonstrated that a core stabilisation programme implemented with young basketball players led to improvements in both 10-metre sprint and vertical jump performance. Rahmat et al. (2014), meanwhile, reported that a core stabilisation programme implemented with children aged 9–12 improved their standing long jump performance.

Studies conducted on young footballers have similarly yielded positive results. Hoshikawa et al. (2013) reported improvements in both sprint and jumping performance following core stabilisation training, which was implemented in addition to football training for 12–13-year-old footballers. Boyacı and Afyon (2017), meanwhile, reported that a 12-week core training programme administered to 20 male footballers with an average age of 13.17 ± 0.86 years resulted in significant improvements in 20-metre sprint, standing long jump and vertical jump performance. Furthermore, Sannicandro et al. (2024) demonstrated that core stability exercises added to warm-up programmes can improve jumping performance in young footballers.

However, the findings regarding jumping performance are not consistent across all studies. A study conducted by Prieske et al. (2022) reported that, whilst core strength training improved sprint performance, it did not result in a significant change in vertical jump height. This suggests that core training may not have the same effect on different components of physical performance.

The varying results in jumping performance can be attributed to a number of factors. Consequently, an increase in core strength alone may not always lead to a significant improvement in jumping performance. Furthermore, the duration and intensity of the training programme, the type of core exercises used, whether the exercises are performed on stable or unstable surfaces, the athletes’ level of development, and the type of jump test used can be considered as possible reasons for the differences in the results. Combining core training with plyometric or lower-body strength training, on the other hand, may lead to more pronounced adaptations in jumping performance.

### General assessment of the findings

In addition to the general trend observed in the literature, the findings of meta-analyses also support the positive effects of core training on sporting performance. Saeterbakken et al. (2022) reported that core strengthening training produces moderate effects on sport-specific performance, effects ranging from small to large on physical fitness, and moderate effects on trunk muscle endurance. These results demonstrate that core training is a complementary training method capable of supporting various performance components.

However, the moderator analyses of the present meta-analysis have shown that the effect of core training on speed and jumping performance did not vary significantly according to certain study characteristics examined. It was found that the effect size tended to decrease as sample size increased for speed performance, whilst it tended to increase as the year of publication progressed. However, the fact that neither correlation is statistically significant indicates that the sample size and year of publication do not provide a meaningful explanation of the effect size relating to speed performance.

With regard to jumping performance, it was observed that the effect size tended to increase as sample size and training duration increased, whilst it tended to decrease as the year of publication progressed. However, it was determined that none of these relationships were statistically significant. It can therefore be said that sample size, year of publication and training duration alone are not sufficient to explain the magnitude of the effect on jumping performance. This suggests that the effect of core training may arise more from the interaction of multiple factors, such as the nature of the training, exercise selection, intensity, sport-specificity and athlete characteristics. From a methodological perspective, the formal Cochrane RoB 2 assessment revealed that the majority of trials exhibited ‘some concerns’, driven predominantly by the unavoidable absence of participant and personnel blinding in exercise interventions, whereas outcome measurement objectivity remained high across timing gates and jump testing systems. Regarding publication bias, while Egger’s test indicated significant asymmetry for speed performance (p = 0.015), trim-and-fill adjustments demonstrated that the overall positive effect direction remained robust (adjusted SMD = 0.686). This asymmetry suggests that small-study effects and true between-study heterogeneity across athletic cohorts, rather than systematic suppression of null findings alone, must be considered when interpreting sprint performance adaptations.

A study conducted on university athletes also suggested that incorporating core stability exercises into training programmes may contribute to the improvement of upper limb performance and that, for this reason, athletes, coaches and strength and conditioning specialists may wish to consider including them as part of their training programmes (Dongaz et al., 2024). This finding suggests that core training can be regarded as a complementary training method not only in terms of lower-limb performance-such as sprinting and jumping-but also in terms of various other components of sporting performance.

Taken as a whole, the existing literature and the findings of this meta-analysis suggest that core training may be beneficial for improving sprint and jumping performance. In particular, a significant proportion of studies conducted in football, basketball, volleyball and other team sports have yielded positive results. However, the fact that no significant improvement was observed in some studies suggests that the effect of core training depends on the specific conditions. The differences between positive and negative results may be related to factors such as the athletes’ age and performance level, training history, the duration and frequency of the programme, choice of exercises, training intensity, the sport-specific nature of the core exercises, and the performance tests used.

Current findings demonstrate that core training yields moderate, statistically significant enhancements in sprint (SMD = 0.601) and jumping (SMD = 0.489) performance across athletic populations. However, it is beyond the empirical scope of this meta-analysis to conclude definitively that combining core training with sport-specific sprint, strength, or plyometric protocols produces superior or synergistic adaptations compared to isolated interventions, as multimodal interactions were not directly modelled as distinct comparative arms. These combined strategies remain plausible physiological hypotheses that warrant direct comparative investigation in future trials. Furthermore, while quantitative subgrouping in the present meta-analysis was constrained by the uneven distribution of studies across disciplines, clinically meaningful factors-including athlete competitive level (elite vs. amateur), sport discipline, training frequency, exercise modality (stable vs. unstable surfaces), and whether core exercises are isolated or integrated into complex conditioning-represent critical potential moderators. Future experimental research should systematically account for these variables to identify optimal athlete-specific prescription guidelines.

## Conclusion

The results of this meta-analysis indicate that core training has generally positive effects on both sprint and jumping performance. With regard to sprint performance, it was found that the effect size tended to decrease as the sample size increased and to increase as the year of publication advanced; however, neither of these relationships was found to be statistically significant. It can therefore be said that the sample size and the year of publication do not explain the effect size on speed performance to a significant degree.

With regard to jumping performance, it was found that the effect size tended to increase as sample size and training duration increased, whilst it tended to decrease as the year of publication progressed. However, it was observed that none of these relationships were statistically significant. Consequently, there is insufficient evidence to suggest that sample size, year of publication and duration of training have a significant effect on the magnitude of the impact on jumping performance.

In general, it can be concluded that core training is a useful and complementary training approach for improving speed and jumping performance. However, it is considered that the magnitude of the effect on performance cannot be explained solely by sample size, year of publication or training duration; factors such as training content, exercise selection, intensity, frequency of application, athlete characteristics and sport-specificity must also be taken into account. In conclusion, core training represents an effective, complementary neuromuscular intervention for enhancing sprint and jumping performance. Future research should prioritize well-controlled trials to determine optimal training dosages, exercise modalities, and potential interactions with other conditioning components across specific athletic tiers.

## Data Availability

The raw data supporting the conclusions of this article will be made available by the authors, without undue reservation.
