# Supplementary material for: The effects of core training on athletic performance (speed and jumping) in different sports: a meta-analysis
Source: Front Physiol. 2026 Oct 2;17:1946447. doi: 10.3389/fphys.2026.1946447 (PMC13672002; doi:10.3389/fphys.2026.1946447)
Supplement: Supplementary file 1 [file Table1.docx]

**Supplementary Material**

**Manuscript Title:** The Effects of Core Training on Athletic Performance (Speed and Jumping) in Different Sports: A Meta-Analysis

**Table S1.** Detailed Database-Specific Search Strategies and Retrieval Syntax

| **Database** | **Detailed Search Syntax / Boolean Strings** | **Date Limits** | **Filters Applied** | **Hits Retrieved** |
| --- | --- | --- | --- | --- |
| **PubMed/ MEDLINE** | ("core training"[Title/Abstract] OR "core exercise"[Title/Abstract] OR "core stability"[Title/Abstract] OR "trunk training"[Title/Abstract] OR "core strengthening"[Title/Abstract]) AND (speed[Title/Abstract] OR sprint[Title/Abstract] OR "sprint performance"[Title/Abstract] OR jump[Title/Abstract] OR "jumping performance"[Title/Abstract] OR "vertical jump"[Title/Abstract] OR "countermovement jump"[Title/Abstract]) | 2020/01/01 to 2026/06/30 | Article | 670 |
| **Web of Science (Core Collection)** | TS=(("core training" OR "core exercise" OR "core stability" OR "trunk training" OR "core strengthening") AND (speed OR sprint OR "sprint performance" OR jump OR "jumping performance" OR "vertical jump" OR "countermovement jump")) | 2020 to 2026 | Article | 13,913 |
| **Google Scholar** | allintitle: ("core training" OR "core exercise" OR "core stability") (speed OR sprint OR jump) | 2020 to 2026 | Articles | 9,450 |
| **Total Records Identified** | — | — | — | 24,033 |

**Table S2.** Cochrane Risk of Bias 2 (RoB 2) Detailed Domain Breakdown across Included Outcome Evaluations (n = 70)

| **Risk of Bias Domain** | **Low Risk (n, %)** | **Some Concerns (n, %)** | **High Risk (n, %)** | **Primary Methodological Drivers- Notes** |
| --- | --- | --- | --- | --- |
| **Domain 1: Bias arising from the randomisation process** | 25 (35.7%) | 41 (58.6%) | 4 (5.7%) | Unclear reporting of allocation sequence concealment in some field sports settings. |
| **Domain 2: Bias due to deviations from intended interventions** | 0 (0.0%) | 64 (91.4%) | 6 (8.6%) | Inherent logistical impossibility of blinding participants and conditioning coaches during active physical exercise protocols. |
| **Domain 3: Bias due to missing outcome data** | 67 (95.7%) | 3 (4.3%) | 0 (0.0%) | Exceptionally high exercise compliance and minimal attrition rates across organized sports cohorts. |
| **Domain 4: Bias in measurement of the outcome** | 63 (90.0%) | 7 (10.0%) | 0 (0.0%) | Use of objective, automated electronic timing photocell gates (sprint) and force/optojump systems (jump). |
| **Domain 5: Bias in selection of the reported result** | 48 (68.6%) | 22 (31.4%) | 0 (0.0%) | Reported outcomes directly corresponded to standard, pre-planned field testing batteries. |
| **Overall Methodological Judgement** | **1 (1.4%)** | **63 (90.0%)** | **6 (8.6%)** | **'Some concerns' category is predominantly driven by unavoidable lack of participant blinding in Domain 2.** |

**Table S3.** Characteristics of Coding and Unit-of-Analysis Prioritization Rules for Outcome Extraction

| **Outcome Category** | **Primary Prioritized Metric** | **Rationale for Statistical Independence** | **Multi-Arm Trial Handling Rule** |
| --- | --- | --- | --- |
| **Speed Performance** | 20-meter sprint split (or nearest intermediate sprint distance) | Represents the optimal kinematic balance between initial linear acceleration and maximum sprint velocity. | When multiple experimental core arms shared a single control group, control group sample size was split proportionally across comparisons to prevent unit-of-analysis inflation. |
| **Jumping Performance** | Countermovement Jump (CMJ) height (cm) | Standardized physiological measure of stretch-shortening cycle (SSC) neuromuscular utilization. | Same proportional sample size allocation applied to control groups across multi-arm intervention designs. |
